# Dual Solid-State PBPK Modelling to Assess the Bio(in)equivalence of Tacrolimus Amorphous Formulations with Storage-Induced Crystallisation: A Potential Application of Virtual Bioequivalence

**DOI:** 10.3390/pharmaceutics18101307

**Published:** 2026-10-15

**Authors:** Kyprianos Michailides, Ali Tabatabaeian Nimavardi, Siri Kalyan Chirumamilla, David B. Turner

**Affiliations:** MID3 Division, Certara UK, Sheffield S1 2BJ, UK; ali.nimavardi@certara.com (A.T.N.); sirikalyan.chirumamilla@certara.com (S.K.C.); david.turner@certara.com (D.B.T.)

**Keywords:** Tacrolimus, Amorphous solid dispersion, PBPK, mechanistic dissolution, virtual bioequivalence

## Abstract

**Background/Objectives**: Amorphous drug products are susceptible to crystallisation during manufacture or storage, which may alter dissolution and pharmacokinetics (PK). Tacrolimus, a low-solubility, high-permeability drug, relies on amorphous formulations to achieve adequate absorption, making crystallisation a key quality risk. Physiologically based pharmacokinetic (PBPK) models with simultaneous tracking of amorphous and crystalline fractions as different solid states within the gut enable a mechanistic assessment of how crystallinity may influence PK and bioequivalence (BE) outcomes. **Methods**: A PBPK model incorporating mechanistic dissolution was developed to evaluate the impact of tacrolimus solid-state composition on BE. Model performance was verified against clinical PK data for formulations spanning a range of crystallinity levels. Virtual BE (VBE) assessments were conducted using two-way and fully replicated four-period crossover designs. Within-subject variability (WSV) was introduced mechanistically through physiological parameters. **Results**: The PBPK model adequately reproduced the crystallinity-dependent PK of tacrolimus observed in clinical studies. VBE simulations captured the reported trend of crystallinity-driven bioinequivalence, with increasing crystallinity primarily influencing C_max_ and having a limited effect on AUC_0–24h_, suggesting a predominant impact on absorption rate. Model-based extrapolation beyond the clinically evaluated range indicated that crystallinity levels of 5–15% may be associated with a higher likelihood of meeting BE criteria under a fully replicated four-period crossover design. **Conclusions**: Mechanistic PBPK modelling enabled extrapolation beyond clinically tested crystallinity levels by linking solid-state behaviour to in vivo performance. This approach can complement experimental evidence and support BE assessment and risk evaluation during formulation development, clinical evaluation, and post-approval change management.

## 1. Introduction

Physiologically based pharmacokinetic (PBPK) modelling integrates physiology and anatomy with drug-specific physicochemical properties to predict absorption, distribution, metabolism, and excretion (ADME) and in vivo performance [1]. PBPK has become a key tool in the pharmaceutical industry for drug product development and lifecycle management and has increasingly contributed to regulatory decision-making and approvals [2,3]. More than two decades ago, physiologically based oral absorption modelling moved beyond representing the gastrointestinal tract as a single compartment, with the introduction of compartmental absorption and transit (CAT) models [4]. Since then, substantial progress has been made, resulting in the emergence of more advanced models, including the nine-segment Advanced Dissolution, Absorption and Metabolism (ADAM) model [1].

The ADAM model combines detailed physiological descriptions of the gut, including population variability, with sophisticated mechanistic algorithms to predict dissolution and absorption of drug products. On the physiological side, for example, it can handle luminal fluid volumes and the fluid volume dynamics after taking a drink or meal in a physiologically realistic manner and can incorporate dynamic bile salt concentrations [5]. Mechanistic approaches enable capturing gut regional differences in pH, bile salts, fluid volumes, and their potential impact on between- and within-subject variability of solubility, dissolution, absorption, and ultimately drug bioavailability. The approach is enabled at least in part through the modelling of in vitro experiments [6,7]. Its utility from a biopharmaceutic perspective has been demonstrated in numerous applications across diverse formulation strategies, including enabling formulations such as salts and amorphous solid dispersions [8,9,10,11,12,13,14,15,16,17]. Mechanistic biopharmaceutic modelling tools are particularly relevant for compounds with dissolution-rate-limited absorption, such as Biopharmaceutics Classification System (BCS) Class II and IV drugs, including tacrolimus.

Tacrolimus is a leading immunosuppressant, with nearly 95% of kidney transplant patients discharged on a tacrolimus-based regimen [18]. As a BCS Class II compound with poor solubility and high permeability, it exhibits dissolution-limited oral absorption. It shows high regional permeability in preclinical perfusion studies (rat jejunum apparent permeability (P_app_) ≈ 1.4 × 10^−4^ cm/s), consistent with its designation as a high-permeability compound, although no direct effective human permeability (P_eff_) measurements are available [19]. The solubility of the crystalline form can be as low as 1.8 µg/mL; hence there is a reliance on solubility-enhancing formulations [20]. Due to its neutral (non-ionisable) nature, it cannot be formulated as a salt to improve solubility and dissolution behaviour. Consequently, the innovator product (Prograf^®^, Astellas Pharma) is an amorphous solid dispersion. Since the approval of Prograf, a number of generic oral products have been developed and approved by the United States Food and Drug Administration (FDA). One such generic, manufactured for Accord Healthcare by Intas Pharmaceuticals Ltd., was found to be susceptible to crystallisation during storage and to exhibit inter-batch differences in the extent of crystallisation [21]. This raises important considerations regarding the impact, if any, of different degrees of crystallisation on BE relative to the innovator product, and the extent of crystallinity at which bioinequivalence may occur.

Previously, these questions were partially addressed through a PBPK modelling approach [20]. In that study, dissolution was characterised by directly inputting a variety of in vitro dissolution profiles into the PBPK model with the assumption that they represent in vivo dissolution. PBPK models were developed and used in virtual BE analysis to estimate the level of crystallinity leading to bioinequivalence. This approach, however, has a number of potential issues and limitations. It is not sensitive to regional or inter-subject differences in physiological parameters such as intestinal fluid volumes, pH, and bile salt concentrations. This lack of sensitivity is particularly important for molecules where dissolution is the rate-limiting step for absorption [7]. In addition, direct input of dissolution profiles lacks extrapolative capability, meaning it cannot reliably predict dissolution at crystallinity levels that have not been tested in vitro or in alternative populations such as paediatrics.

In the present study, rather than using in vitro dissolution profiles to represent in vivo dissolution, mechanistic models of dissolution are applied, including a diffusion layer model (DLM), a particle population balance model, and explicit tracking of two solid states of the drug (amorphous and crystalline). By explicitly linking dissolution to gut physiology, this approach is expected to be sensitive to regional and inter-subject variability and to retain extrapolative capability beyond tested conditions, addressing the limitations described above. In addition to these advantages, this approach also removes the need to select, perhaps somewhat arbitrarily, a suitable in vitro dissolution profile. Rather, the DLM can be evaluated and parameterised across a range of in vitro dissolution experiments prior to being used to simulate in vivo dissolution. The modelling results are compared with clinical data from studies where the dosage form contained various proportions of crystalline and amorphous material. This integrated approach may enable the prediction of BE limits for crystallisation with greater confidence and accuracy than direct input of in vitro dissolution profiles into PBPK models.

## 2. Methods

### 2.1. Materials

The Simcyp Simulator (version 25, release 1; MID3, Certara UK, Sheffield, UK) (the Simulator) was used to model and predict the in vitro dissolution and in vivo clinical PK of tacrolimus. The in vitro dissolution profiles of Prograf and Accord formulations, used to parameterise the mechanistic DLM in the Simcyp^®^ In Vitro Data Analysis toolkit (SIVA, Version 5; MID3, Certara UK Limited, Sheffield, UK), were obtained from Purohit et al. (2018) [20]. Clinical PK data used for model verification and virtual bioequivalence (VBE) analysis were obtained from Taylor et al. (2025) [22]. An overview of the model development and its application in VBE is provided in Figure 1.

### 2.2. Absorption

Oral absorption of tacrolimus was described using the ADAM model. For parameterising gut wall permeability, tacrolimus-specific permeability data from standard in vitro assays such as Caco-2, MDCK/MDR1 efflux, or PAMPA passive permeability assays are limited. Therefore, permeability obtained from rat intestinal perfusion experiments was used as the basis for parameterisation. Tamura et al. (2002) developed a correlation between rat in situ single-pass intestinal perfusion permeability and human in vivo effective permeability, using a set of reference compounds with previously determined values for both parameters [19]. In this study, the intrinsic transcellular permeability (*P*_trans,0_) in the mechanistic permeability (MechPeff) model was optimised to match the P_eff_ of 4.77 × 10^−4^ cm/s estimated by Tamura et al. (2002) [19]. MechPeff is a mechanistic model built into Simcyp that predicts passive effective intestinal permeability (P_eff_) across different gut regions by combining transcellular, paracellular, and mucus layer permeabilities, using regional gut physiology and morphology, and drug physicochemical properties [23].

To model solubility, aqueous solubility data were obtained from the literature, with amorphous solubility reported at approximately 0.05 mg/mL [24]. Biorelevant solubility data from the literature [25] were then used to determine the bile micelle:buffer partition coefficient (K_m:w_) using the SIVA toolkit. K_m:w_ values are used in solubility calculations to represent bile salt micelle-mediated solubilisation effects. The estimated logK_m:w_ values were very similar for crystalline tacrolimus (4.52, 90% CI: 4.4–4.6) and amorphous tacrolimus (4.62, 90% CI: 4.5–5.5). Given the close agreement between these values and the predicted logK_m:w_ value of 4.70 based on the equation developed by Glomme et al. (2006) [26], the latter value was applied in the model for both solid states.

To model dissolution, the mechanistic particle population balance (PPB) model was utilised, as it explicitly tracks dispersion of particle populations across gastrointestinal regions while preserving count and mass balance; in the Simulator, the PPB model is required to enable use of the two-solid-state model. This approach allows the undissolved amorphous and crystalline solid states of tacrolimus to be parameterised independently. A mechanistic DLM was used instead of directly entering in vitro dissolution data and arbitrarily selecting the most suitable profile. The DLM is based on the Wang and Flanagan (2002) model, which predicts drug dissolution by assuming a hydrodynamic boundary layer around drug particles through which diffusion is the rate-limiting step [27]. The model accounts for drug-specific factors such as particle size and solubility, as well as prevailing physiological conditions within the gastrointestinal tract. The DLM was parameterised in SIVA 5 by modelling in vitro dissolution profiles in 40, 100 and 450 mL of United States Pharmacopeia (USP) test dissolution medium I (pH 4.5 aqueous medium containing 50 µg/mL hydroxypropyl cellulose) from Purohit et al. (2018) [20]. The modelling required the estimation of empirical correction factors, referred to as DLM scalars [7], for each solid state of both Prograf and the generic formulation [20].

### 2.3. Distribution

For distribution, the Simulator parameter estimation (PE) tool was used to fit a minimal PBPK model to intravenous infusion data [28]. Clinical blood concentrations were first converted to plasma concentrations prior to performing the PE procedure. The first-order rate constants describing the transfer of tacrolimus into (k_in_) and out of (k_out_) the single adjusting compartment (SAC), along with the Kp scalar used to adjust the predicted tissue-to-plasma partition coefficients (Kp values), were optimised.

### 2.4. Concentration-Dependent Blood-to-Plasma Ratio

Tacrolimus exhibits extensive and saturable binding to erythrocytes, such that a fixed blood-to-plasma (B/P) ratio may not adequately capture its distribution between plasma and red blood cells across the concentration and haematocrit ranges encountered clinically; this behaviour has been shown to influence predicted exposure across a range of haematocrit values and dose levels [29]. To account for this, the concentration-dependent B/P ratio approach described by El-Khateeb et al. [29] was incorporated into the model. Unlike a fixed B/P ratio, the concentration-dependent function reduces the need for compensatory parameter adjustments and is expected to improve the physiological realism of the PBPK predictions. The average haematocrit of 0.557 for the liver transplant patients was back-calculated from the observed data [30]. Blood binding estimates were obtained using this value and the observed concentration data. These parameters were then used to generate concentration-dependent B/P ratios assuming a standard haematocrit of 0.45, and the resulting profile was implemented in the Simulator (Table S1).

### 2.5. Elimination

Tacrolimus elimination was described using recombinant human cytochrome P450 (rhCYP) data [31], with the intersystem extrapolation factor (ISEF) applied as a scalar. The ISEF values were optimised using the same intravenous infusion data [28]. The model input parameters can be found in Table 1.

### 2.6. Clinical Verification

Clinical verification of the PBPK model was performed using the observed PK data [22]. The study evaluated Prograf and an amorphous generic tacrolimus formulation (Accord), as well as the same generic formulation subjected to stress conditions to achieve two levels of crystallinity, approximately 20% and 50% crystalline tacrolimus in the dosage form. Whole blood concentration–time profiles over 24 h were used to verify model predictions for both C_max_ and AUC_0–24h_ across all four formulations.

### 2.7. VBE Design

The VBE simulations were informed by the single-dose, four-treatment, four-period crossover study described by Taylor et al. (2025) [22], in which 24 healthy male and female volunteers aged 18–49 years received a 5 mg dose under fasting conditions and completed all four periods. As in the clinical BE analysis, pairwise two-way comparisons were made, with Prograf as the reference and the amorphous and crystalline generic formulations as test products. Standard BE acceptance limits of 80–125% were applied to the 90% confidence interval of the geometric mean test/reference ratio for C_max_ and AUC_0–24h_. Ten virtual trials of 24 subjects were simulated per formulation. The model was also extrapolated to a fully replicated four-period crossover design, as recommended in FDA regulatory guidance for narrow therapeutic index (NTI) drugs [35].

To account for WSV in the VBE analysis, a mechanistic approach was adopted. WSV was introduced into physiological parameters identified as influential through sensitivity analysis. Where available, default coefficient of variation (CV) values in the Simulator, which were obtained by meta-analysis of physiological data from repeated measurements in the same subject, were used. For parameters for which no meta-analysis data were available, a CV of 30% was assigned (Table S2).

## 3. Results

### 3.1. Mechanistic Modelling of In Vitro Dissolution

In vitro–in vivo extrapolation (IVIVE) of dissolution and release kinetics for different solid states (i.e., amorphous and crystalline) can be challenging due to limitations in the transferability of in vitro experiments to the in vivo setting, particularly in the presence of precipitation. For Prograf, which showed no evidence of precipitation or crystallisation, a more straightforward approach was applied. Dissolution data obtained using two media volumes (40 mL and 100 mL) were modelled in SIVA 5, and a single DLM parameter was optimised to describe the dissolution behaviour [20]. The global DLM scalar for solid state 1 (amorphous) was estimated to be 0.349, yielding an acceptable fit to the observed dissolution data (AAFE = 1.05, based on 16 data points) (Figure 2).

For the fresh generic formulation, the amorphous fraction was set to 100%. In vitro dissolution experiments indicated that tacrolimus precipitation commenced after approximately 30 min [20]. Therefore, only dissolution data from the initial 30 min were used for parameter optimisation. The global DLM scalar for solid state 1 was estimated to be 1.16, yielding satisfactory agreement with the observed dissolution data (AAFE = 1.05, based on 8 data points) (Figure 3).

For parameterisation of the crystalline fraction of the generic formulation, dissolution data from the 100% crystalline formulation obtained in 100 and 450 mL media were used. Data from the 40 mL medium were excluded because early drug precipitation was observed, making parameterisation unreliable. The previously reported solubility of the crystalline state was 0.0018–0.0149 mg/mL depending on the dissolution media [20]. Therefore, this parameter was optimised to 0.0038 mg/mL to match the extent of dissolution, expressed as the maximum concentration dissolved, observed in the two dissolution media volumes used for parameterisation. The global DLM scalar for solid state 2 (crystalline) was subsequently estimated to be 1.69, demonstrating satisfactory agreement with the observed dissolution data (AAFE = 1.10, based on 16 data points) (Figure 4).

### 3.2. Clinical Verification of Prograf and Generic Formulations at Different Crystallinity Levels

Drug-specific parameters were kept constant across all the formulations evaluated in the verification stage. These included Prograf, the 100% amorphous generic formulation, and generic formulations containing 20% and 50% crystalline material. The only distinction between the Prograf^®^ and generic formulation models was the DLM scalar associated with solid state 1 (amorphous). This adjustment accounted for formulation-specific differences in dissolution behaviour while maintaining a consistent underlying physiological framework. For the generic formulations containing crystalline fractions, the same model structure was retained, with variation introduced solely through the proportion of each solid state to reflect the formulation’s degree of crystallinity. The 20% crystalline formulation was represented by 80% solid state 1 and 20% solid state 2, while the 50% crystalline formulation was represented by equal contributions from both solid states. As illustrated in Figure 5, these limited and mechanistically justified modifications were sufficient to reproduce the distinct PK profiles observed with increasing crystalline content, without the need for additional parameter adjustments.

As shown in Table 2, the predicted-to-observed ratios for C_max_ and AUC_0–24h_ were within the predefined 80–125% acceptance limits for all the formulations. For Prograf, T_max_ was underpredicted (predicted/observed ratio = 0.57). Although the mechanistic dissolution model captured a delay in dissolution relative to the amorphous generic, this delay resulted in a simulated increase in T_max_ from 0.96 to 1.13 h, which remained lower than the observed T_max_ of approximately 2 h. Conversely, for the 50% crystalline generic, T_max_ was overpredicted relative to the observed data.

### 3.3. VBE Assessment and Crystallinity-Driven Bioinequivalence Thresholds

A PBPK-based VBE assessment was conducted to evaluate whether the model could reproduce the crystallinity-dependent bioinequivalence patterns observed clinically for tacrolimus formulations under fasting conditions. In the published clinical study, the amorphous, 20% crystalline, and 50% crystalline generic formulations failed to demonstrate BE to Prograf. Bioinequivalence was primarily driven by differences in C_max_, while AUC_0–24h_ remained within BE limits for the 20% and 50% crystalline generics [22]. For the 100% amorphous generic, the simulated VBE trials showed elevated C_max_ ratios relative to Prograf, with the 90% confidence intervals from many virtual trials exceeding the upper BE limit of 125%, as shown in Figure 6. For the 20% crystalline generic, the simulated C_max_ ratios frequently approached or fell below the lower BE boundary, mirroring the clinical outcome in which C_max_ BE was not demonstrated despite equivalence in AUC_0–24h_. The PBPK simulations reproduced this selective sensitivity of C_max_ to partial crystallinity, while AUC_0–24h_ ratios remained within acceptance limits across most trials. For the 50% crystalline generic, simulated C_max_ ratios were consistently and markedly lower than those of Prograf, with all the virtual trials falling well below the lower 80% BE limit. This closely aligns with the observed clinical geometric mean ratio of approximately 56% for C_max_. In contrast, simulated AUC_0–24h_ point estimates (geometric mean ratios) remained within the 80–125% acceptance limits, although the corresponding confidence intervals extended beyond these limits in several trials.

The model was then used to explore BE across additional crystallinity levels. A high likelihood of BE was defined as at least 80% of trials (i.e., 8 out of 10) falling within the 80–125% acceptance range. As shown in Figure 7, crystallinity levels of approximately 10% were associated with an 80% probability of being bioequivalent to the reference product.

It should be noted that these results are specific to the single-dose, four-treatment crossover design used by Taylor et al. (2025) [22], simplified here into pairwise two-treatment comparisons for the VBE simulations. These findings may differ under a fully replicated four-period crossover design, which is recommended in FDA regulatory guidance for narrow therapeutic index (NTI) drugs [35]. In the VBE simulations using a fully replicated crossover design, generic formulations with crystallinity in the range of 5–15% were predicted to meet FDA BE requirements in at least 80% of the simulated trials, whereas the fully amorphous generic formulation (0% crystallinity) met the C_max_ criterion in only 50% of trials, reflecting its faster dissolution and absorption rate and consequently higher C_max_ (Figure 8).

## 4. Discussion

The developed PBPK model reproduced the key clinical trends in tacrolimus PK and BE across formulations. This performance was supported by the incorporation of a non-linear concentration-dependent blood-to-plasma ratio, which is critical for accurately capturing the saturable erythrocyte binding behaviour of tacrolimus. The model also provided mechanistic insight into the role of the degree of crystallinity in absorption. Notably, the relationship between crystallinity and C_max_ BE was not linear. The fully amorphous generic, which dissolved faster than Prograf, showed a reduced likelihood of meeting the C_max_ criterion, whereas modest crystallinity (c. 10%) improved this likelihood by bringing the formulation’s dissolution rate closer to that of Prograf. At higher crystallinities, C_max_ was progressively reduced, an effect that became markedly more pronounced at 50% crystallinity, where C_max_ was reduced substantially relative to Prograf, whereas AUC_0–24h_ geometric mean ratios were less affected. Collectively, these results support the inference that increasing crystallinity primarily affects the rate, rather than the extent, of tacrolimus absorption.

Precipitation of tacrolimus within the gastrointestinal tract was not parameterised in the model, based on evidence that such precipitation is unlikely to occur in vivo. The nucleation induction time of tacrolimus in buffer was reported as 340 min, which is longer than the average gastrointestinal transit time [36]. Additionally, both formulations contain hydroxypropyl methylcellulose (HPMC), which can act as a precipitation inhibitor and stabilise the solution at the amorphous concentration limit. The only evidence contradicting this rationale comes from in vitro dissolution experiments. However, since these were conducted in the absence of an absorptive component (e.g., a dissolution-permeation system) or transit (e.g., multiple vessel system), they may overestimate the true in vivo precipitation risk [20]. This is illustrated in Figure 9: the simulated in vivo dissolution profiles rapidly achieved complete dissolution, whereas the corresponding in vitro profiles [20,22] for the generic peaked within the first 30–60 min before declining, consistent with supersaturation-driven precipitation within the closed, non-absorptive in vitro system. For formulations containing a crystalline fraction, the simulated in vivo profiles displayed two distinct phases: an initial rapid phase governed by dissolution of the amorphous fraction, followed by a slower phase corresponding to dissolution of the crystalline fraction, consistent with the markedly lower aqueous solubility of the crystalline form.

Despite this overall performance across all the tested formulations for AUC_0–24h_ and C_max_, some discrepancies in the predicted T_max_ were observed. The underprediction of T_max_ for Prograf may reflect limitations in the model’s representation of capsule disintegration and dissolution kinetics, or may partly arise from the clinical sampling schedule, which could limit the precision with which T_max_ was estimated. The opposite discrepancy observed for the 50% crystalline generic, where T_max_ was overpredicted, may similarly be attributed to incomplete characterisation of the dissolution behaviour of the crystalline fraction within the model, or to the same constraints imposed by the clinical sampling schedule.

For the BE assessment, tacrolimus is designated as an NTI drug [37]. Regulatory guidance for NTI products recommends a fully replicated four-period crossover design to support reference-scaled average bioequivalence (RSABE) evaluation, since this design allows the WSV of the reference product to be characterised and enables simultaneous assessment of both systemic exposure and variability between test and reference formulations [35]. The clinical study reported by Taylor et al. (2025) [22] employed a two-way crossover design that does not conform to this recommended regulatory standard and should therefore be regarded as exploratory, providing preliminary evidence ahead of a more comprehensive BE investigation.

In the VBE simulations, WSV was introduced mechanistically, through physiological parameters, rather than by superimposing the observed intra-subject CV (ISCV) of PK parameters from a prior clinical trial onto simulated PK parameters (post hoc WSV); the latter approach was avoided as it is less mechanistic and offers limited extrapolative capability [38]. Because both model structure and WSV were represented mechanistically, the resulting PBPK model could be extrapolated with greater confidence to a fully replicated four-period crossover design, providing a more regulatory guidance-compliant estimate of the crystallinity range likely to satisfy FDA BE requirements for an NTI drug. Under this framework, two formulations are considered BE if: (i) the 95% upper confidence bound for the RSABE criterion is less than or equal to zero; (ii) the conventional unscaled average BE criterion is met, such that the 90% confidence interval for the test-to-reference geometric mean ratio lies within 80–125%; and (iii) the upper limit of the 90% equal-tailed confidence interval for the ratio of within-subject standard deviations does not exceed 2.500, as specified in FDA draft guidance for Abbreviated New Drug Application (ANDA) submissions [35].

Beyond the ability of the model to predict crystallinity-driven bioinequivalence thresholds, some aspects of the underlying model parameterisation warrant caution. Tacrolimus is a known substrate of the efflux transporter P-glycoprotein (P-gp), and the underlying rat perfusion data were generated in the absence of a P-gp inhibitor, so any P-gp-mediated efflux is inherently lumped into the rat-derived P_eff_ value. Where rat-human differences in P-gp expression exist, the resulting estimate of human Peff may therefore be unreliable. A related source of uncertainty concerns the fraction of tacrolimus unbound in gut enterocytes (fu_Gut_), a sensitive parameter for which no experimental data are available. As tacrolimus undergoes extensive enterocyte metabolism [39], fu_Gut_ has a direct effect on predicted intestinal availability (F_g_). However, different approaches to estimating this parameter led to an overprediction of the reported F_g_ [40,41]. The default Simcyp value of 1 was therefore retained, reflecting the absence of a mechanistically justified alternative rather than a validated estimate, which represents a further limitation of the enterocyte metabolism component of the model.

## 5. Conclusions

This study illustrates how mechanistic PBPK modelling can be used to explore formulation-related BE risks beyond conditions directly tested in vitro or clinically. By explicitly linking solid-state properties, dissolution, and gastrointestinal physiology to systemic exposure, the approach allows cautious extrapolation to untested scenarios and supports interpretation of the observed BE outcomes. More broadly, mechanistic models can complement experimental data across multiple stages of drug development. These include formulation optimisation, clinical study design, and post-approval change assessment. Together, they provide a transparent and scientifically grounded framework for decision-making when empirical data are limited.

## Figures and Tables

**Figure 1:**
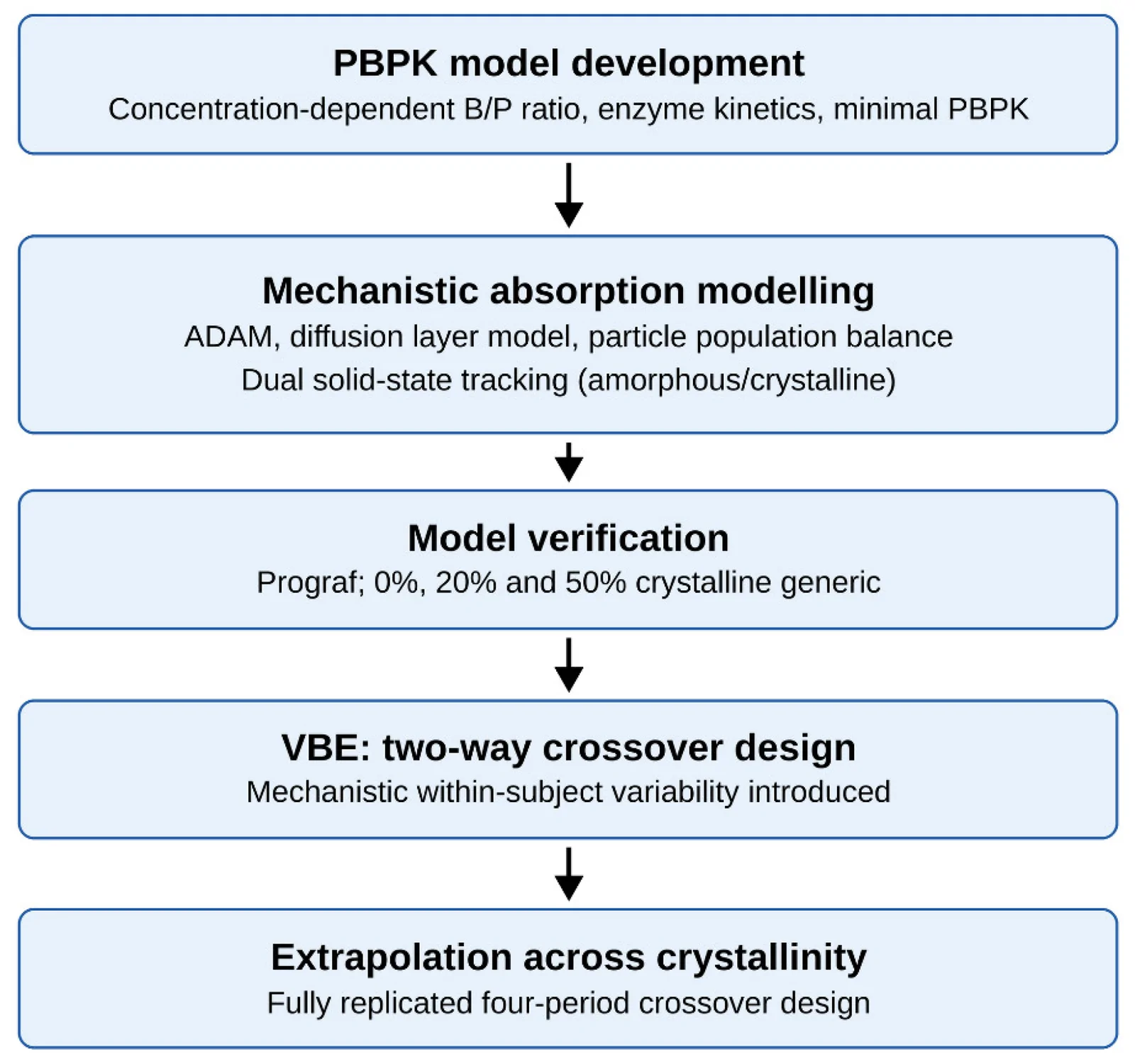
Overview of the PBPK model development and its application in VBE.

**Figure 2:**
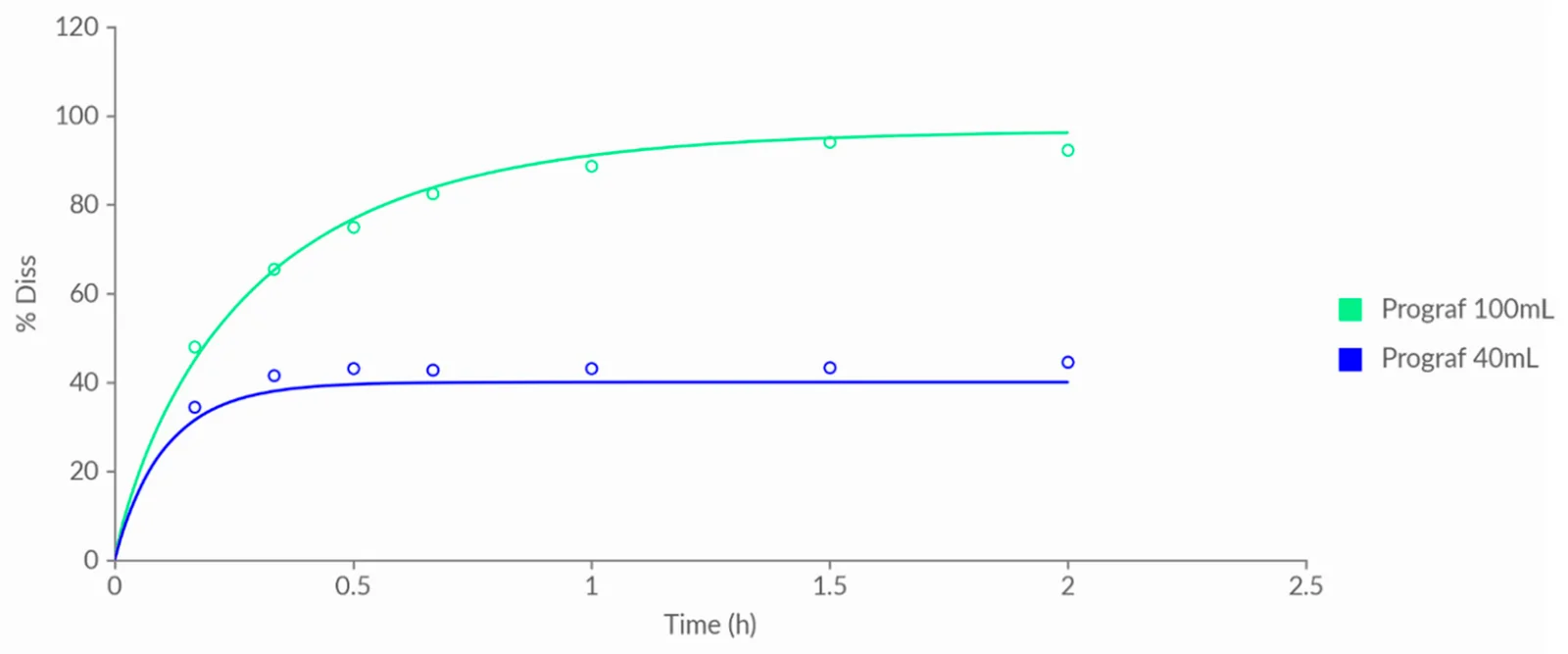
DLM scalar for solid state 1 optimisation for Prograf in SIVA 5 using dissolution data from Purohit et al. (2018) [20].

**Figure 3:**
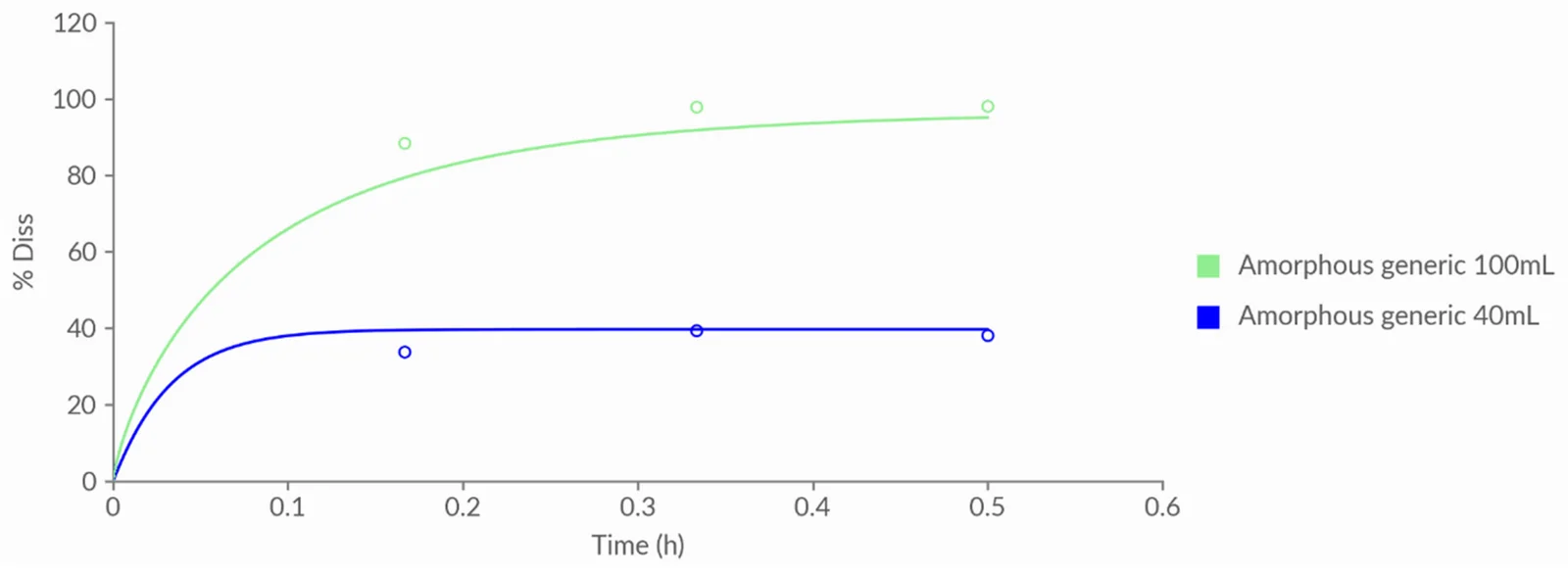
DLM scalar for solid state 1 optimisation for the generic formulation in SIVA 5 using dissolution data from Purohit et al. (2018) [20].

**Figure 4:**
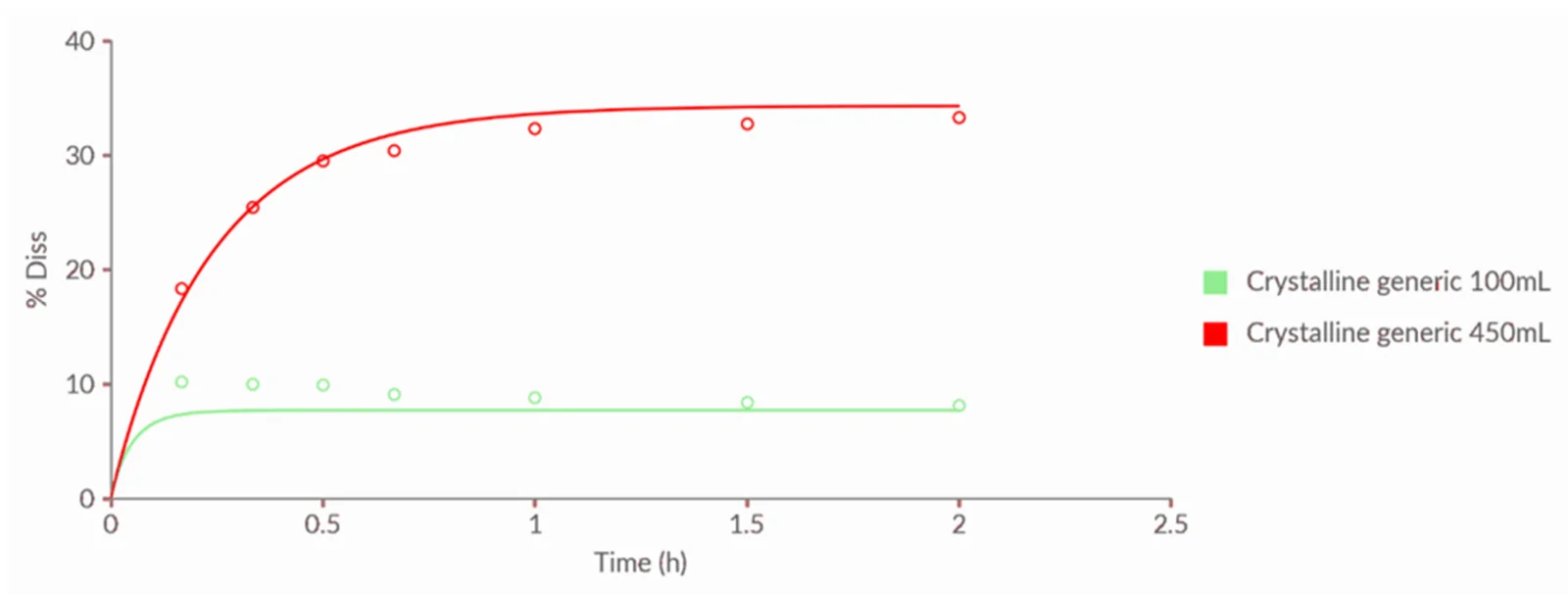
DLM scalar for solid state 2 optimisation for the generic formulation in SIVA 5 using dissolution data from Purohit et al. (2018) [20].

**Figure 5:**
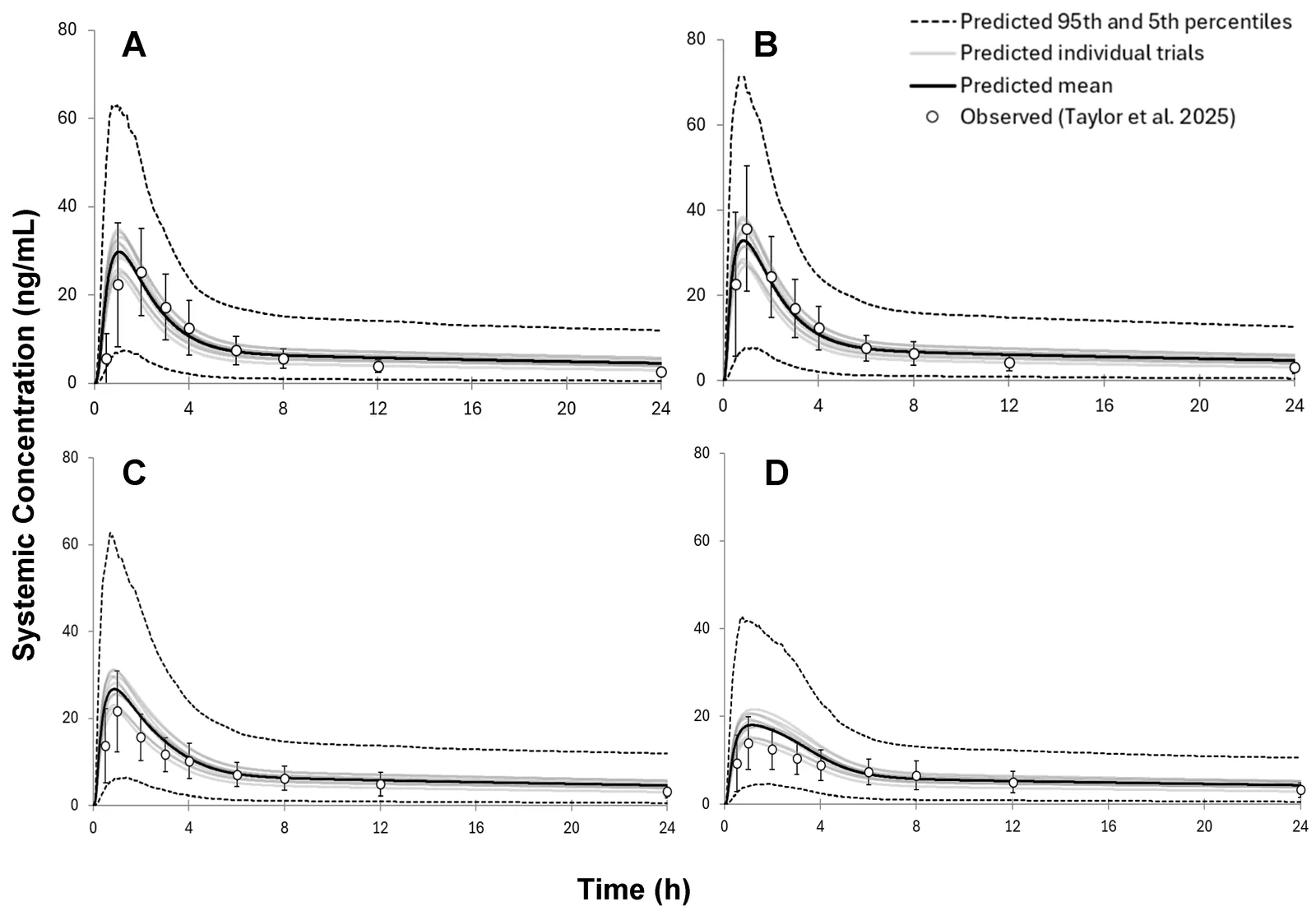
Predicted mean (black line) and observed mean (open circles, error bars represent ±1 SD, *n* = 24) concentration–time profiles [22] following oral administration of 5 mg tacrolimus capsules: Prograf^®^ (**A**), amorphous generic formulation (**B**), and generic formulations containing 20% (**C**) and 50% (**D**) crystalline drug. Grey lines represent the predicted individual trials; dashed lines represent the predicted 5th and 95th percentiles.

**Figure 6:**
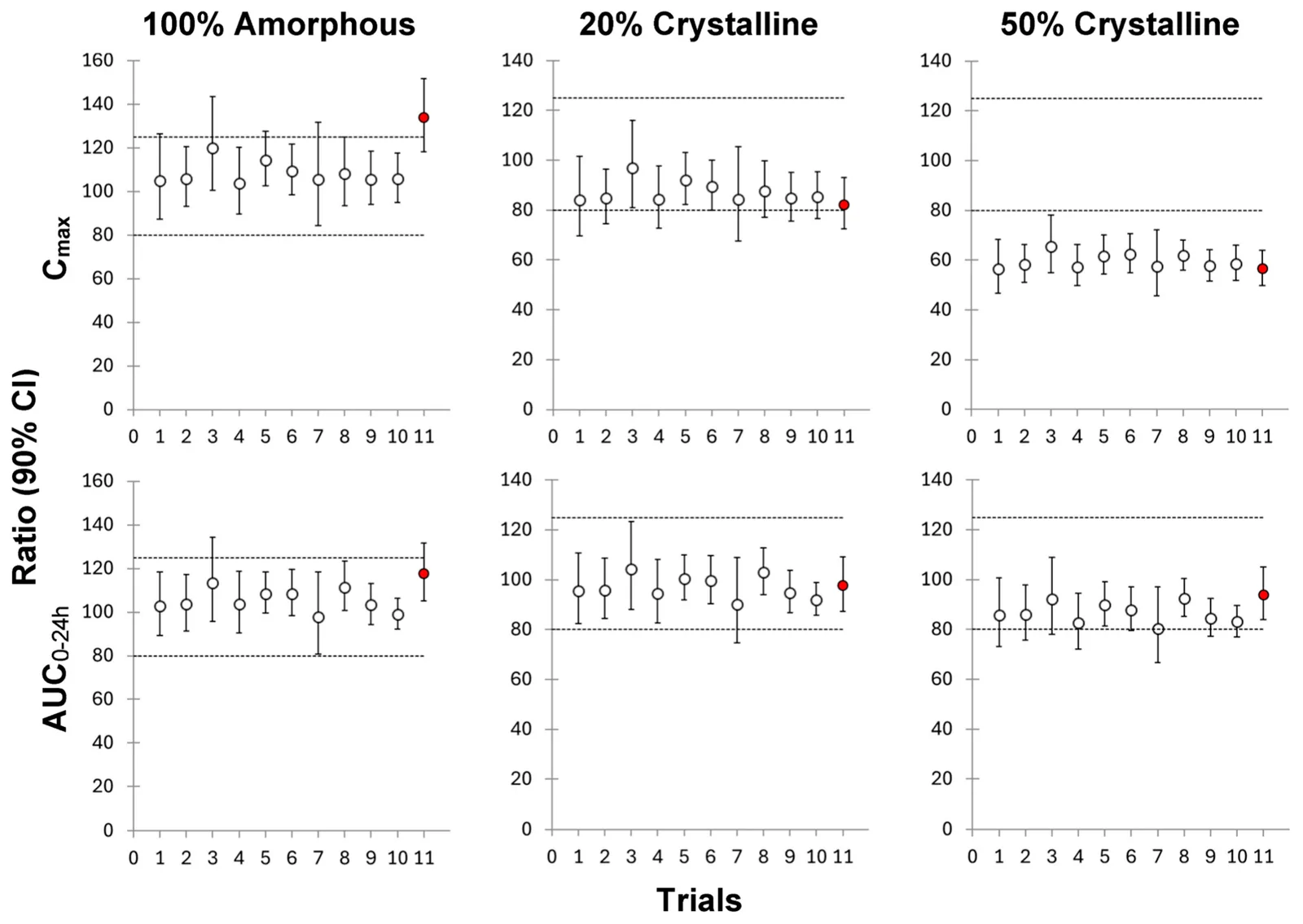
Three sets of two-way crossover VBE simulations were performed in 24 healthy volunteers to evaluate AUC_0–24h_ and C_max_. Fresh Prograf (reference product) was compared with 100% amorphous, 20% crystalline, and 50% crystalline generic formulations. Ten virtual trials were simulated per set, alongside one observed clinical dataset (red dot) reported by Taylor et al. (2025) [22]. Error bars represent the 90% confidence intervals for the PK metrics. The dotted lines indicate the BE acceptance limits of 80–125%.

**Figure 7:**
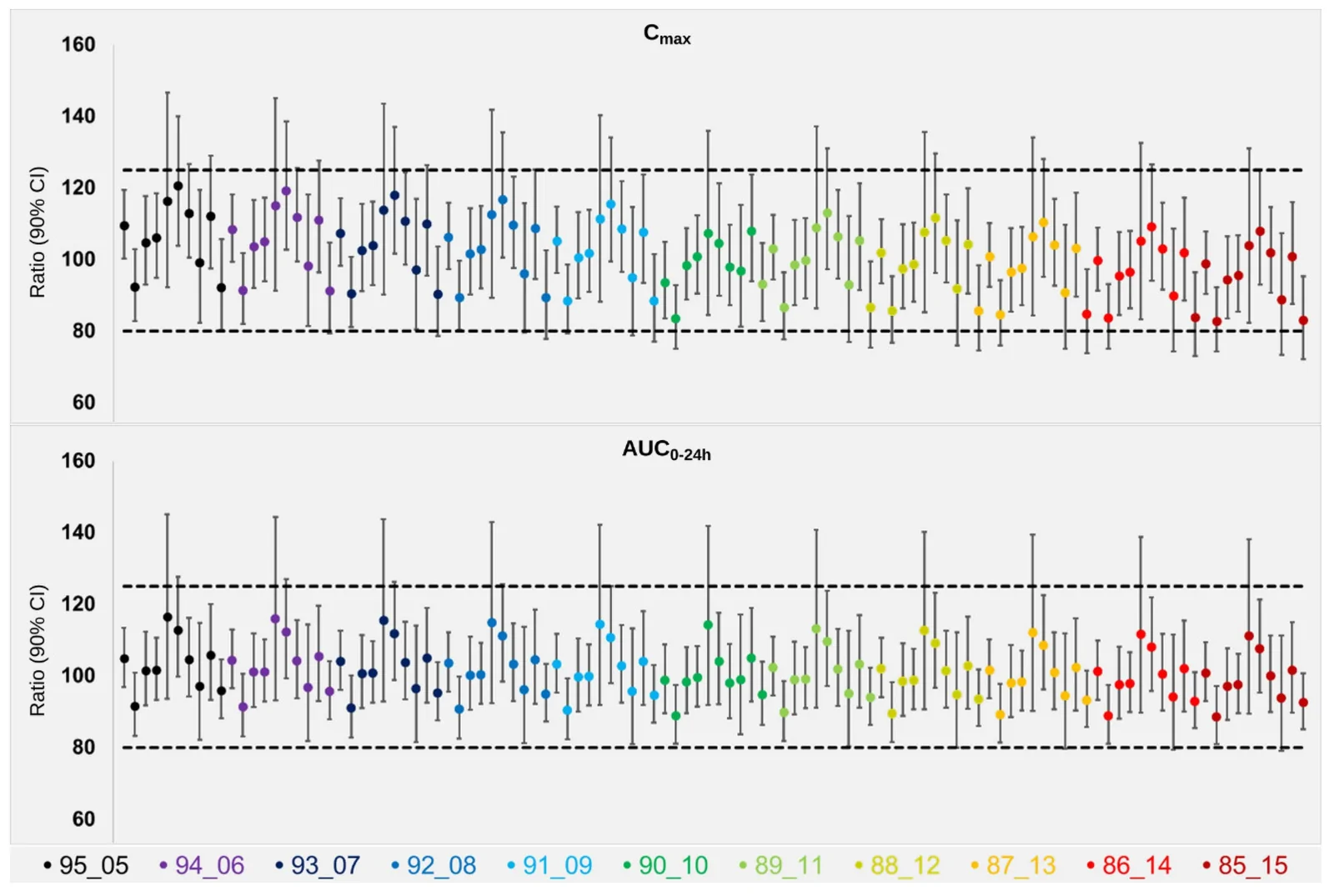
Two-way crossover VBE extrapolation simulations conducted in 24 healthy volunteers to evaluate AUC_0–24h_ and C_max_ across crystallinity levels ranging from 5% to 15%. For each crystallinity level, ten virtual trials were simulated. Legend labels indicate the solid-state composition as % amorphous_% crystalline (e.g., 95_05 represents 95% amorphous and 5% crystalline tacrolimus). Data points represent the geometric mean test-to-reference ratios, with error bars indicating the 90% confidence intervals (CIs) for the PK metrics. Dashed horizontal lines denote the BE acceptance limits of 80–125%.

**Figure 8:**
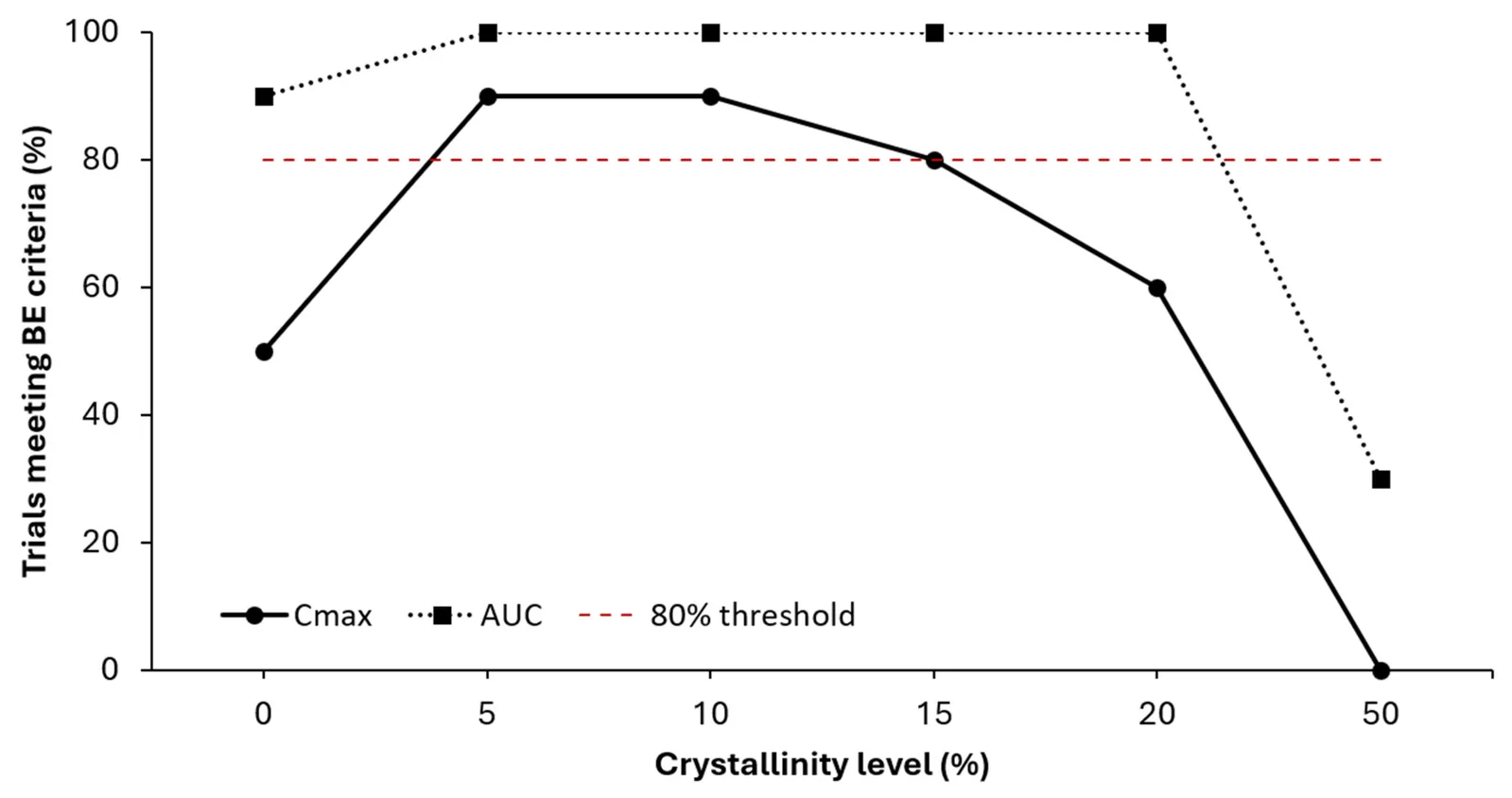
Percentage of virtual trials meeting BE criteria per PK metric in a fully replicated four-period crossover design.

**Figure 9:**
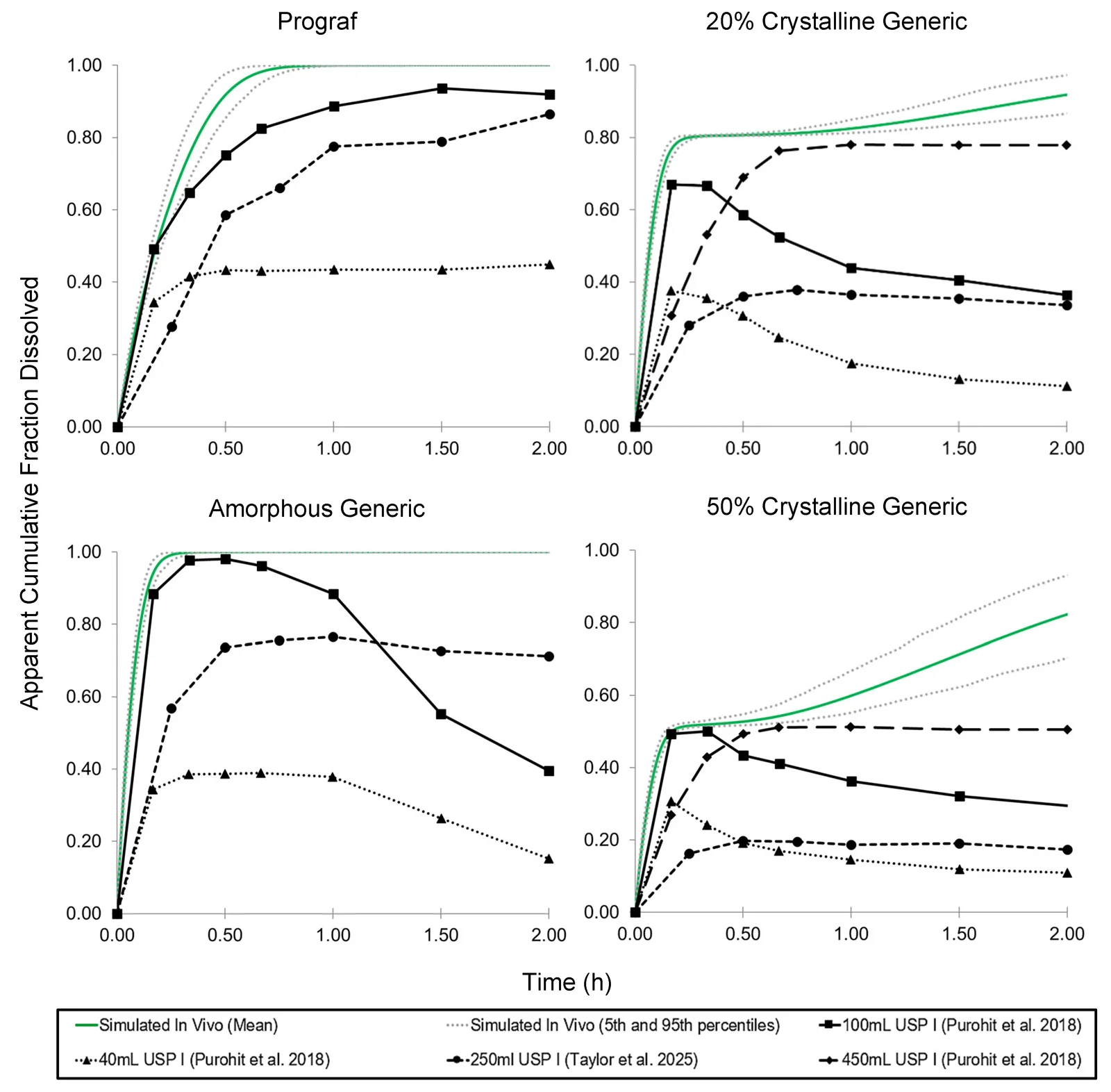
Simulated in vivo cumulative dissolution compared with in vitro dissolution profiles of Prograf, amorphous generic, and 20% and 50% crystalline generics [20,22].

**Table 1:** Simcyp PBPK input parameters.

Parameter	Value	Comment/Reference
**Physicochemical Properties**		
Molecular weight (g/mol)	804.02	[32]
LogP_o:w_	3.26	[33]
Compound Type	Neutral	
**Blood Binding Properties**		
B/P Ratio	Concentration-dependent profile	[29,30]
fu	0.013	[34]
**Absorption–Permeability**		
Model	ADAM	
fu_Gut_	1	Simcyp default
P_trans,0_ (×10^−6^ cm/s)	54.42	Optimised to reproduce reported Jejunum I Peff,man [19]
P_eff,man_ (×10^−4^ cm/s)	4.77	
**Distribution Parameters**		
Model	Minimal PBPK	
Vss (L/kg)	4.6955	Method 2 with fitted Kp scalar: 4.2575 Optimised using IV profile from [28]
Vsac (L/kg)	1 × 10^−5^	
k_in_ (1/h)	0.70284	
k_out_ (1/h)	0.11234	
**Elimination Parameters**		
Enzyme	V_max_/Km	
CYP3A5–12-HT	1.4/0.35	[31]
CYP3A4–12-HT	0.6/0.29	
CYP3A5–13-DMT	17/0.21	
CYP3A4–13-DMT	8/0.21	
ISEF	0.65	Optimised using IV profile from [28]

LogP_o:w_, logarithm of the octanol-water partition coefficient; B/P, blood-to-plasma ratio; fu, fraction unbound in plasma; ADAM, Advanced Dissolution, Absorption and Metabolism model; fu_Gut_, fraction unbound in the gut; Peff,man, human jejunal effective permeability; MechPeff, mechanistic permeability model; P_trans,0_, intrinsic transcellular permeability; Vss, volume of distribution at steady state; Vsac, volume of the single adjusting compartment (SAC); Kp, tissue-to-plasma partition coefficient; k_in_, first-order rate constant for transfer into the SAC; k_out_, first-order rate constant for transfer out of the SAC; ISEF, intersystem extrapolation factor; 12-HT, 12-hydroxytacrolimus; 13-DMT, 13-O-demethyltacrolimus; CYP3A4/CYP3A5, cytochrome P450 3A4/3A5; Vmax (pmol/min/pmol), maximum reaction rate; Km (μM), Michaelis-Menten constant; IV, intravenous.

**Table 2:** Predicted and observed PK parameters for tacrolimus 5 mg capsule formulations.

		Observed	Predicted	Pred/Obs
Prograf	AUC_0–24h_	156 ± 45	193 ± 130	1.24
	T_max_	2 (1.0–4.0)	1.13 (0.5–2.3)	0.57
	C_max_	29 ± 12	31 ± 18	1.06
Amorphous generic	AUC_0–24h_	184 ± 61	207 ± 140	1.12
	T_max_	1 (0.5–3.0)	0.96 (0.3–1.9)	0.96
	C_max_	38 ± 14	34 ± 21	0.91
Generic 20% crystalline	AUC_0–24h_	154 ± 51	189 ± 127	1.23
	T_max_	1 (0.5–3.0)	1.01 (0.4–3.1)	1.01
	C_max_	23 ± 8.5	28 ± 18	1.22
Generic 50% crystalline	AUC_0–24h_	147 ± 46	167 ± 109	1.14
	T_max_	1 (0.5–8.0)	1.40 (0.4–3.7)	1.40
	C_max_	15.6 ± 4.9	19 ± 12	1.23

Values are mean ± SD, except T_max_, which is median (range). AUC_0–24h_, ng·h/mL; T_max_, h; C_max_, ng/mL. Pred/Obs, ratio of predicted to observed mean.

## Data Availability

Data are contained within the article and supplementary materials.

## Supplementary Materials

The following supporting information can be downloaded at https://www.mdpi.com/article/10.3390/pharmaceutics18101307/s1. Table S1: Concentration-dependent blood-to-plasma ratio; Table S2: Selected within-subject variability (WSV) parameters.
