# Supplementary material for: Dual Solid-State PBPK Modelling to Assess the Bio(in)equivalence of Tacrolimus Amorphous Formulations with Storage-Induced Crystallisation: A Potential Application of Virtual Bioequivalence
Source: Pharmaceutics. 2026 Oct 15;18(10):1307. doi: 10.3390/pharmaceutics18101307 (PMC13708303; doi:10.3390/pharmaceutics18101307)
Supplement: Supplementary file 1 [file pharmaceutics-18-01307-s001.zip › pharmaceutics-4585950-supplementary.pdf]

# Supplementary Materials

## *Supplementary Materials Table S1 Concentration-dependent blood to plasma ratio*

| Conc. (mg/L) | 0.000001 | 0.0107 | 0.02788 | 0.031481 | 0.041024 | 0.046511 | 0.07789 | 0.095361 | 0.1159 | 0.14732 |
|---|---|---|---|---|---|---|---|---|---|---|
| B/P | 58.069 | 53.601 | 46.467 | 44.973 | 41.024 | 38.759 | 25.963 | 19.072 | 11.59 | 4.2092 |

## *Supplementary Materials Table S2 Selected Within Subject Variability (WSV) parameters*

| ***Selected Parameter*** | *Variation (%CV)* |
|---|---|
| *Bile Fasted Stomach* | 213 |
| *Bile Fasted Duodenum* | 30 |
| *Bile Fasted Jejunum 1* | 77 |
| *Bile Fasted Jejunum 2* | 77 |
| *Bile Fasted Ileum 1* | 30 |
| *Bile Fasted Ileum 2* | 30 |
| *Bile Fasted Ileum 3* | 30 |
| *Bile Fasted Ileum 4* | 30 |
| *Stomach Basal Volume (mL)* | 48 |
| *Basal Water Volume in Total SI Segment (mL)* | 29 |
| *Mean Gastric Emptying Time Fasted* | 38.217 |
| *Mean Small Intestinal Transit Time* | 21.132 |
| *Fasted MRT Stomach Fluid* | 30 |
| *Fasted MRT SI Fluid* | 30 |
| *Cardiac Output Male* | 30 |
| *Cardiac Output Female* | 30 |
| *Duration of IMMC Cycle from Antrum (h)* | 30 |
| *Duration of IMMC Cycle from Duodenum (h)* | 30 |
| *Fasted Gallbladder Residual Volume (%) - IMMC start from Antrum* | 30 |
| *Fasted Gallbladder Residual Volume (%) - IMMC start from Duodenum* | 30 |
| *Fasted-Total Hepatic Bile Salts Mass Rate (mmol/h)* | 30 |
| *% of Hepatic Bile Entering Gallbladder* | 30 |
